# Raptor Models Alter Rodent Behavior During Acorn Dispersal

**DOI:** 10.1002/ece3.74630

**Published:** 2026-11-01

**Authors:** Sergio Del Arco, Jose María Del Arco

**Affiliations:** ^1^ Faculty of Biology University of Salamanca Salamanca Spain; ^2^ Department of Agroforestry Sciences Higher Technical School of Agrarian Engineering of Palencia Palencia Spain; ^3^ iuFOR Sustainable Forest Management Research Institute, University of Valladolid Valladolid Spain

**Keywords:** acorns' dispersal, acorns' fates, foraging behavior, raptor models, rodents' activity

## Abstract

The relationship between rodents and oak trees has attracted considerable attention. The interaction spans a wide range of outcomes, from extreme predation, in which rodents consume acorns, to forms of collaboration that approach mutualism. Acorn characteristics and the behavior of rodent species within the oak habitats are among the factors shaping this interaction. This study examines the influence of third species on rodent–oak interaction by using raptor models. The main objective is to determine whether raptors in the treetops can deter rodents from feeding on acorns, thereby reducing acorn dispersal. To achieve this objective, we monitored acorns' dispersal by four rodent species, Algerian mouse, wood mouse, common vole, and garden dormouse, in 10 m × 10 m semi‐wild enclosures with oak trees in the four corners, two of them with raptor models. Beneath the 4 oaks, 60 acorns, 15 acorns per tree, were provided daily for 15 days, and the acorns in situ consumption and dispersal were tracked to determine whether the presence of raptor models altered the dispersal by any of the rodent species. The results show that species that are actively involved in acorn dispersion through transport (Algerian mouse, wood mouse) modify their behavior in response to the presence of raptor models in the treetops. The garden dormouse also modifies its behavior, although this species does not transport or bury acorns; however, it does move them around on the surface and consumes most of the acorns whole by preying on them. Therefore, it does not participate in the acorn dispersion process. The common vole typically occupies open habitats without oaks and does not modify its behavior, probably because raptor models are not perceived as a threat.

## Graphical abstract

The presence of birds of prey in the branches of oak trees reduces the ability of rodents to disperse acorns.

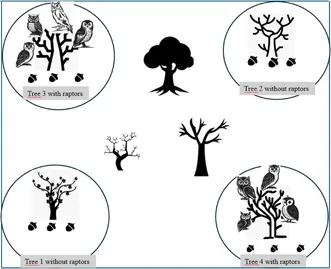

## 1 Introduction

Research on the interaction between rodents and oak trees has attracted attention for several decades (Zhang and Zhang 2008; Mittelman et al. 2021; Zhang et al. 2025). Early studies focused mainly on predatory interactions in which rodents eat acorns (Galetti et al. 2015). Later, it was shown that some rodent species contribute to acorn dispersal by transporting and burying acorns in different types of storage sites (S. B. Vander Wall 1990; Muñoz and Bonal 2011; Yi and Wang 2015; Gu et al. 2017; Lichti et al. 2017), including caches (Zhang et al. 2025; Hou et al. 2024), and larders (Cao et al. 2016; Wang and Corlett 2017). The balance between both predation and dispersal can determine the nature of the interaction between rodents and oak trees, because there is a continuum ranging from the extreme of predation, a hostile relationship for oaks, to the extreme of mutualism, in which both species involved derive benefits, even if these benefits are not symmetrical for both species. It is necessary to determine whether predators alter rodent behavior and shift the relationship to positions far from collaboration. Zwolak and Crone (2012), Chang and Zhang (2014), Xiao and Krebs (2015), Gleditsch et al. (2017), and Bogdziewicz et al. (2020) developed mathematical/energy models to determine whether the balance of costs and benefits in rodent–oak interactions is closer to predation or mutualism.

Rodent–oak interactions should be studied for specific species pairs, since each interaction may have unique characteristics that are not present in other combinations (Zwolak et al. 2020). Moreover, even the same species pair may display different behaviors when the interaction is studied in different regions of the world. This interaction, initially considered predatory, has been shown to shift toward collaboration with oak trees since some rodents participate, to a varying degree, in acorn dispersal (Zhang et al. 2015, 2016; Gómez et al. 2019; Bogdziewicz et al. 2020; Moore and Dittel 2020; Zwolak et al. 2020). Some authors go further and suggest that certain rodent–oak interactions may approach mutualism (Zwolak and Crone 2012; Chang and Zhang 2014; Wang, Cao, and Zhang 2014; Wang, Chen, and Corlett 2014; Xiao and Krebs 2015; Gleditsch et al. 2017; Wróbel and Zwolak 2017).

Numerous factors affect and influence the outcome of these interactions (Wang, Chen, and Corlett 2014; Wróbel and Zwolak 2017). One key aspect is the behavior of rodent species (Zhang et al. 2024). Rodents may consume acorns or leave them uneaten, consume them wholly or partially (Steele et al. 1993), retain or discard the embryo, consume them in situ, transport them, or abandon them on the surface. They may also engage in hoarding behavior, building larders and caches (Hou et al. 2024), and display behavior to prevent theft from stores (Steele et al. 2011; Cheng et al. 2025). Among the most common species in the Iberian plateau, Common voles— *Microtus arvalis* Pallas—do not typically consume acorns or inhabit oak forest areas. This species arrived recently, in the 1980s (Luque‐Larena et al. 2013). In contrast, Algerian mouse— *Mus spretus* Lataste—and wood mouse— *Apodemus sylvaticus* L.—inhabit oak forests and actively scatter their seeds (Gray et al. 1998; Lalis et al. 2019; Del Arco et al. 2018; Del Arco and Del Arco 2022, 2025). Prior observations indicate that garden dormouse— *Eliomys quercinus* L.—consume acorns, but it is unclear how they process them. This species is known to inhabit oak forests, although it can also be found in open grasslands, like the common vole (Fedyń et al. 2021). These behaviors suggest that different rodent species participate to varying degrees in the dispersal of acorns and, therefore, in their interactions with oak trees (Zhang et al. 2015, 2016; Gómez et al. 2019; Bogdziewicz et al. 2020; Moore and Dittel 2020; Zwolak et al. 2020).

Acorn characteristics may also influence these interactions (S. B. Vander Wall 2010; Wang, Cao, and Zhang 2014; Snell et al. 2019; Steele 2021). For instance, acorns may be easy or difficult for rodents to access (thin or thick shells). They may or may not be nutritious or contain chemical deterrents such as tannins (Zhang et al. 2013, 2018; Wang et al. 2020). Clearly, the interactions between rodents and oak trees may produce a wide range of outcomes.

Among the factors influencing rodent–oak interactions, the presence of third‐party predators has received little attention, despite its potential impact on rodent foraging (Sunyer et al. 2013; Mulungu and Martin 2024). Raptors often stalk rodents from the lower branches of the oak trees while they search for acorns during the acorn season. This may deter rodents from foraging beneath tree canopies, reducing their participation in acorn dispersal.

With the aim of exploring the effect of raptor species on rodent–oak relationships, and with the hypotheses that raptor models simulate raptors perched in oak trees, as perceived by rodents, within this study, we selected four rodent species that occupy different habitats and display distinct acorn‐handling behaviors to achieve the following objectives:

1. To determine whether different rodent species avoid the trees with raptor models.
2. To quantify the effect of the raptor models on foraging acorns.
3. To assess the effects of raptor models on acorn early‐stage dispersal to other aboveground locations and storage in caches and larders, with different understory vegetation that provide shelter.

To address these objectives, we selected four rodent species with different behaviors handling acorns, Algerian mouse ( *Mus spretus* ), wood mouse ( *Apodemus sylvaticus* ), common vole ( *Microtus arvalis* ), and garden dormouse ( *Eliomys quercinus* ), quantified in situ consumption and tracked the early‐stage movement and storage of holm oak acorns in four semi‐natural enclosures, differentiating acorns provided beneath oak trees with and without raptor models to determine whether acorn dispersal is reduced under canopies occupied by raptor models. This was done sequentially by rodent species, with camera traps monitoring rodent visits during the 15‐day periods.

## 2 Materials and Methods

In autumn 2024, we selected four 100 m^2^ plots (10 × 10 m) that replicate the experimental design in an oak forest dominated by *Quercus ilex* and *Quercus faginea* near Palencia (Spain). The plots were enclosed using a metal fence to exclude ungulates. To prevent rodent escape, the interior perimeter was lined with 2 m high sheet metal, with 50 cm buried vertically to inhibit the rodents from tunneling out of the enclosure. The rest of the sheet metal, 1.5 m high, protruded above the ground to prevent rodents from escaping or intruding into the enclosure by jumping over it; particularly, the wood mouse, which can jump very high due to its well‐developed hind limbs.

We preserved the natural vegetation structure within the plots, and we disregarded raking the entire plot because it would disturb the habitat strongly. Within each of the four plots, several specimens of *Quercus ilex* and *Quercus faginea* were randomly distributed (25–35 individuals of each species, ~60 trees/plot, with a total of 222 trees within the 4 plots). The tallest trees reached approximately 3 m in height, while the smallest were approximately 70 cm tall and formed part of the understory. Due to this density, their trunks are slender (13.1 ± 2.9 cm in diameter) and modest height (2.06 ± 0.15 m). The average crown diameter is 1.98 ± 1.01 m, and the average height at which the first branches appear is 1.03 ± 0.06 m. At this density, rodents could not see the plot edges or beyond the surrounding trees from any position within the enclosure. Within each plot, one tree was selected from each of the four corners, choosing the individual closest to the corner to maximize the distance between selected trees. Under the canopy of these four trees, all grass, stones, branches, and roots that could provide shelter for the rodents were removed. This area was circular in shape and extended from the trunk to the edge of the canopy. In this clear area, acorns were placed 30 cm from the trunk, as if they had recently fallen from the tree (Figure 1). We decided to clear this circular area beneath all the selected trees to prevent the vegetation from providing cover for the rodents, which would have made it impossible for us to observe the effect of raptor models. In this way, the rodents are forced to venture out into an unprotected area if they wish to feed on the acorns.

**FIGURE 1:**
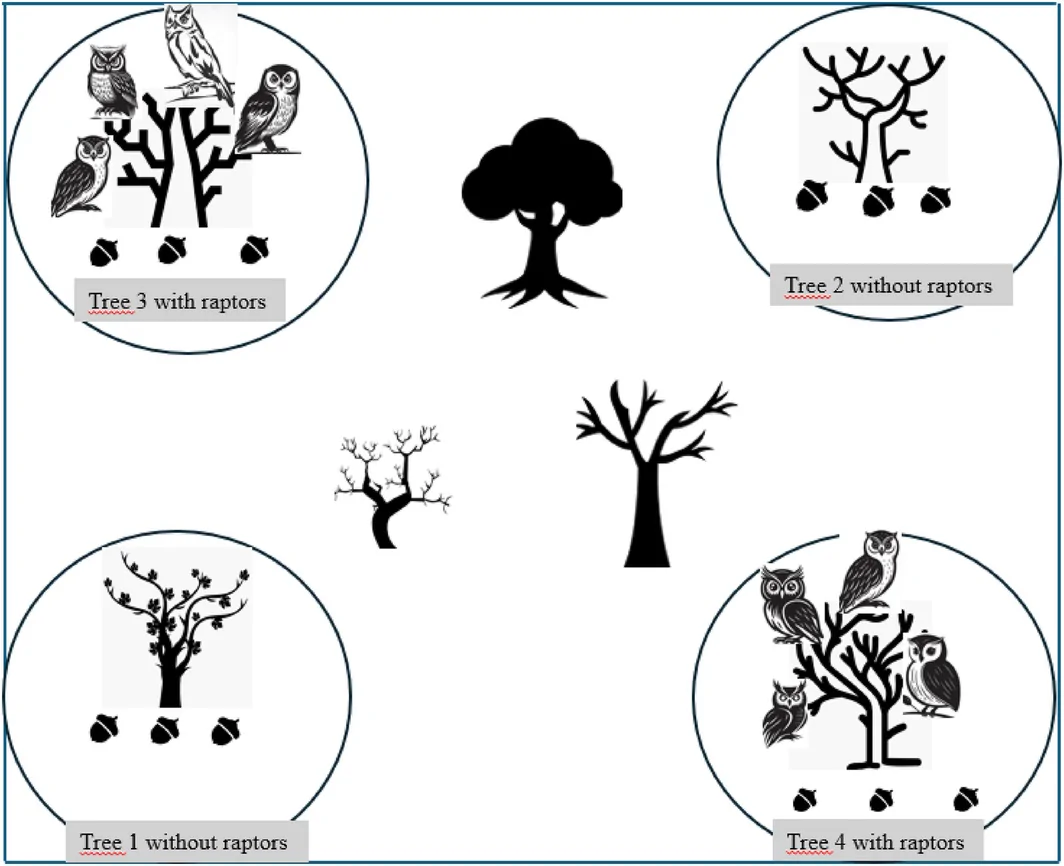
Diagram showing the layout of the four 100 m^2^ plots in which we placed specimens of each rodent species. The circles represent the clear circular areas beneath the canopies of the four trees situated at the four corners. We placed raptor models in the canopies of Trees 3 and 4. Trees 1 and 2 have no raptor models. The vegetation in the rest of the plot is shown schematically. Each plot contained approximately 60 trees distributed randomly within it.

The acorn species chosen was *Quercus ilex* , because it is the dominant species in this forest, and in the region. It is also the species preferred by rodents (Del Arco et al. 2018; Del Arco and Del Arco 2022). Before being placed under the trees in an orderly fashion to facilitate the detection of missing acorns, each acorn was labeled with a plastic tag bearing an identification number. The tag was attached with a wire that pierced the cotyledons in the center of the acorn. These tags permitted the tracking of the origin of each acorn (i.e., the tree from which it was placed) and their final destinations after use.

In each plot, two of the four trees selected at opposite diagonal corners were chosen to hold models of nocturnal and diurnal birds of prey ( *Bubo bubo* L. and *Falco peregrinus* Tunstall) on branches close to the ground (0.5–1.5 m; Figure 2). On each tree, we placed three models of nocturnal raptors and one model of a diurnal raptor, positioned in all directions so that the rodents would spot them from any approach. The models were placed on the lower branches but suspended by strings from the upper branches so that they would be moved by the wind and create a sense of movement. Nocturnal raptor models were included because all four rodent species are nocturnal feeders, while the common vole is also active during the day. To account for this diurnal activity, we also placed models of diurnal raptors. We have preliminary, unpublished data showing that the wood mouse identifies these models of birds of prey as dangerous and distinguishes them from similar objects, but we have not conducted these tests with other species, so we cannot be certain that they identify them as birds of prey. However, Beránková et al. (2014) and Antonová et al. (2021) already evidenced that passerine birds reacted to predatory birds of prey. The four models on the same tree expose rodents to excessive risk, but this was necessary so rodents could detect the models from any direction. Therefore, the experiment likely represents a very strong predator‐cue treatment and may show an upper‐bound response rather than a typical field response to natural raptor presence.

**FIGURE 2:**
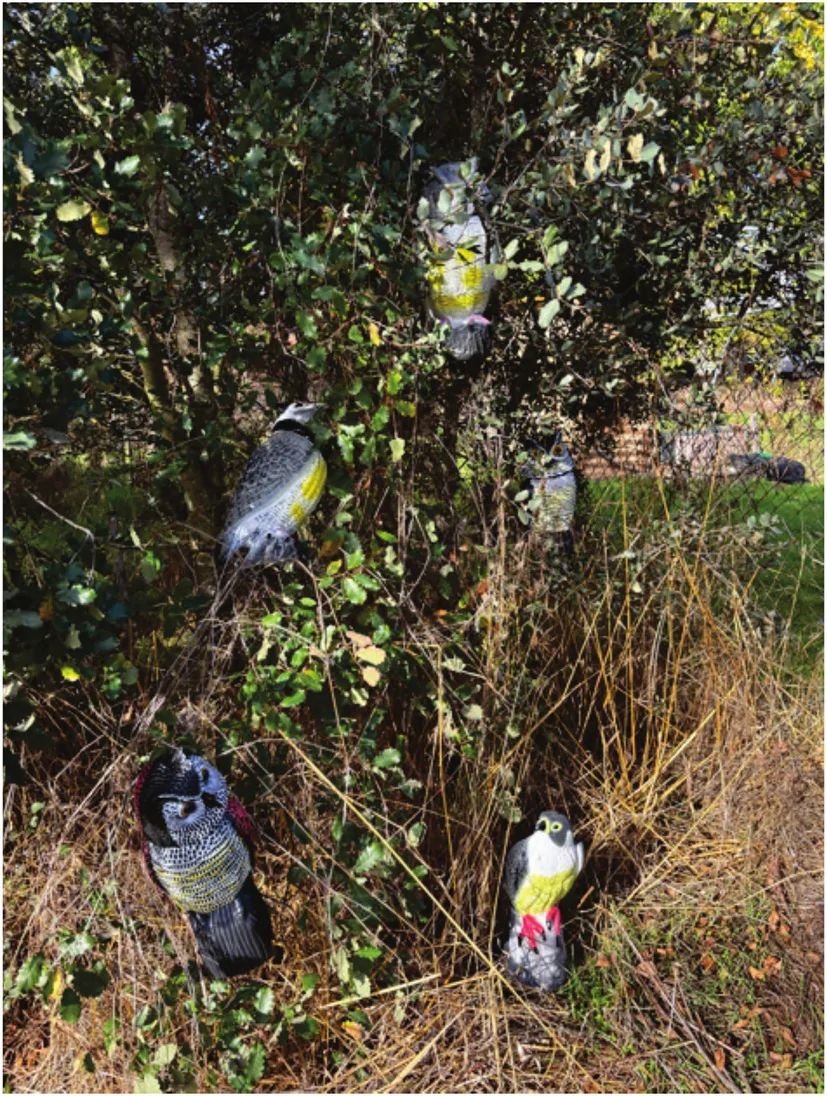
Raptor models before their definitive deployment on the top of the two trees located at the two diagonal corners of each plot in the four semi‐wild plots.

After the plots were prepared, we captured wild specimens of four rodent species (Algerian mouse, wood mouse, common vole, and garden dormouse) in an oak forest dominated by *Quercus ilex* and *Quercus faginea* near Palencia (Spain), as well as in adjacent grasslands and crops. We also conducted trapping sessions over the days prior to the experiment, using Sherman traps, to capture any rodent present within the enclosures to ensure that they were not already occupied by resident rodents. Individuals belonging to the selected rodent species were later incorporated into the experiment, within another plot.

In each of the 4 selected plots, we introduced 4 individuals of the same species: 1 male and 3 females, 16 individuals of each species. Introducing two males in the same plot can lead to competitive interactions, which in some species may result in aggression or death. All the individuals used were adults, although their precise age cannot be determined. A rotational design was implemented for the use of the plots. The sequence began with the wood mouse, which was the first species for which sufficient specimens were captured, followed by the common vole, Algerian mouse, and finally the garden dormouse. The entire experiment took place during the autumn, from September to the first few days of December. During autumn, temperatures are mild with no marked fluctuations, and rainfall is moderate and occurs on random days. For this reason, we do not believe there were any differences in the environmental conditions to which each species was subjected. The vegetation structure of each plot was identical for all rodent species.

The number of plots was limited due to restrictions in the public study area, which prevented the installation of additional enclosures. This small number of plots may limit our ability to explain the results since we can only perform four replicates. The experiment for each species lasted 15 days. At the end of this period, the individuals occupying the plots were recaptured. Before introducing a new species, the plots were left empty for several days to allow the scent marks from the previous occupants to dissipate, and the artificial containers were washed and sterilized with alcohol.

Every day, 60 labeled acorns were provided to the rodents in each plot. They were distributed by placing 15 acorns beneath each of the 4 selected trees, 2 with raptor models and 2 without them. Over the 15‐day experiment period, this amounted to 900 acorns per plot, and a total of 3600 acorns per rodent species. Every day, the number of acorns used was recorded, identifying their location and replacing them. Prior experience indicates that acorns have several possible fates: consumption in situ, abandonment on the ground surface, burial in small groups a few centimeters below the soil surface or under leaf litter, known as caches (S. B. Vander Wall 1990; Hou et al. 2024), and storage of larger numbers of acorns at a greater depth (10–20 cm), in galleries, resting burrows, or larders (S. B. Vander Wall 1990). Consumption in situ; some species of rodents, because of their small size, cannot eat whole acorns; they do not fit in their stomachs, so they leave behind partially eaten remains that, if the embryo is still intact, can germinate. Therefore, this stage is also part of the acorn dispersal process. Some acorns are moved centimeters or meters from their original location and left on the surface, as if the rodents were seeking sheltered places in which to eat the acorns. Other acorns are carried over greater distances to shallow underground storerooms, caches, or deep larders. In all these places, the acorns may be intact, partially eaten, or completely eaten, identifiable to the remains of the husk that are not ingested. As rodents are unlikely to excavate natural larders in 15 days, we simulated these food stores by burying cylindrical plastic containers (20 cm in diameter). The containers were fitted with screw‐on lids to allow access to the interior and to facilitate control of the number of acorns deposited inside. The inside of the containers was connected to the ground surface using an 8 cm diameter rubber hose (Figure 3). These were used in previous works and have been shown to be effective for storing acorns that have been moved frequently. The containers were buried in areas protected by scrub and in open, unprotected locations. For the remaining acorn locations aboveground and for caches, we recorded whether they occur under vegetation cover or in exposed areas. This allows us to assess whether rodents preferred sheltered locations, most likely to reduce predation risk from raptors.

**FIGURE 3:**
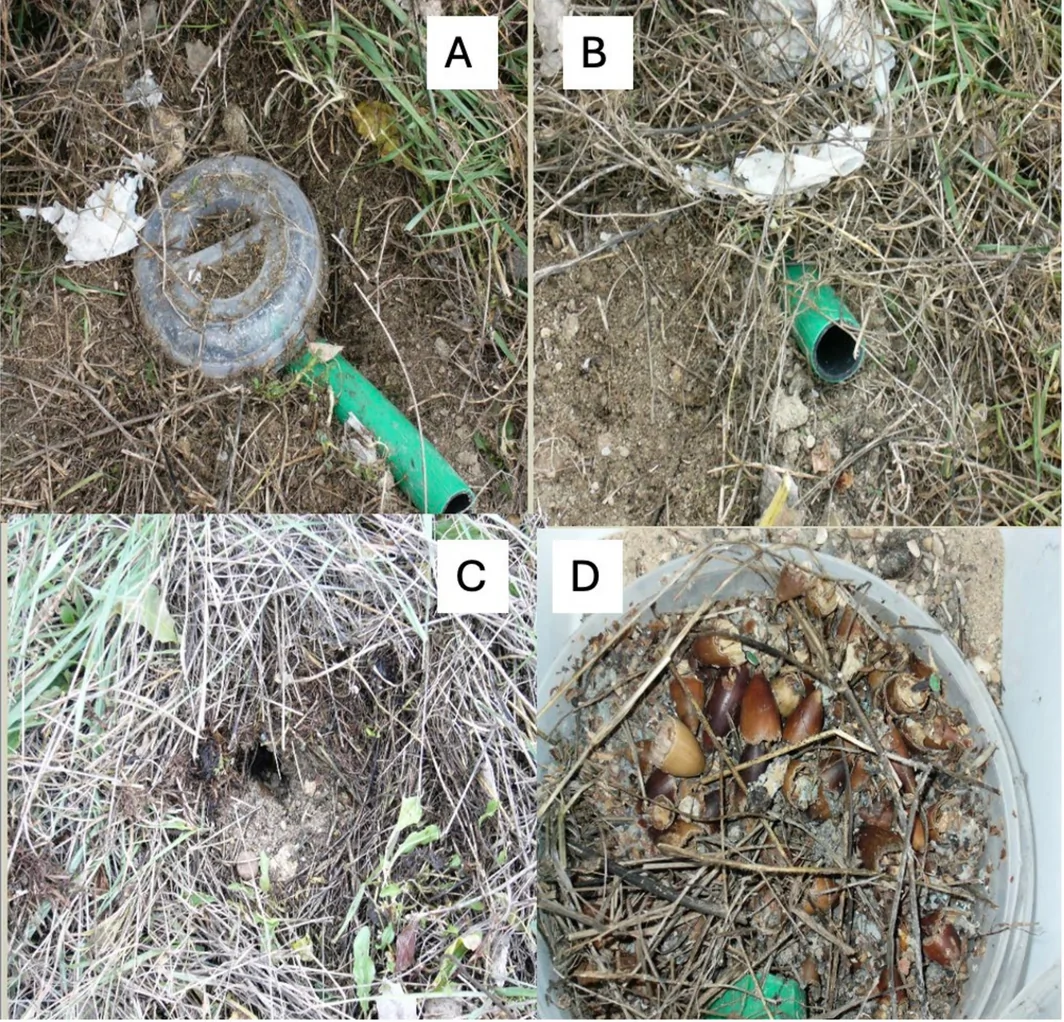
Artificial containers that mimic rodent larder. (A) Cylindrical container with a screw‐off lid, not buried, and tube/pipe connecting the interior to the surface. (B) Buried container with entrance through the tube/pipe. (C) Natural burrow made by Algerian mouse specimens. (D) Container without lid showing the acorns it contains.

Camera traps (Bushnell Nature View CamHDMax) were set up to monitor rodent visits to the canopies covering the acorns. The number of records per tree and rodent species was registered and used as a proxy of raptor model avoidance, since it depends on the number of visits, time spent beneath the trees, and activity within the spot.

### 2.1 Statistical Analyses

The effect of the raptor models on foraging acorns was tested by the analysis of the variance of the generalized linear mixed model (GLMM) of the number of acorns used out of the 15 provided daily per tree. We used binomial distribution with logit link function to account for the two possible outcomes of the acorns: (a) used (consumed or dispersed) or (b) not used (neither consumed nor dispersed). The fixed effects included the main effects of raptor models, rodent species, and experimental day and their interactions. The random effects were the tree ID nested within the plot and the slope of time with the rodent, to account for the dependence among observations within each plot and tree.

The acorns used each day of the experiment constitute repeated measures. The replicates are the four plots occupied by specimens of the same species. The two trees of each type present per plot are considered pseudoreplicates. Since the observations are not entirely independent, the relationships among them are specified through random effects, with the tree effect nested within the plot.

Similarly, we used generalized linear mixed models (GLMMs) to assess the acorn fates, those including in situ consumption and early‐stage dispersal (moved aboveground or stored in caches and larders). The number of acorns within each fate out of the 225 acorns provided during the whole experiment per tree and rodent species followed a binomial distribution with a logit link function.

The main effects and interaction of raptor models, rodent species, and fates were introduced as fixed effects. The random effects were the tree ID, nested within the plot. The time was not used as a fixed or random effect due to the very low occurrence of certain fates.

Model diagnostics were performed through diagnostic plots (i.e., Pearson residuals vs. fitted values; Bates et al. 2015) and homogeneity (Bartlett 1937) and overdispersion tests (Gelman and Hill 2007). Wald's (Type II) Chi‐squared test was performed to get the significance of the GLMM fixed effects.

Multiple comparisons with Benjamini–Hochberg correction were used as a test a posteriori for the significant effects detected for acorns used and their early fates (Benjamini and Hochberg 1995) and identify groups of that are not significantly different. All statistical analyses were implemented in the R software environment (version 4.6.4; R‐Core Team 2025). Using the *lme4* package for GLMM (version 1.1–35.5; Bates et al. 2015), *car* package for Wald's Chi‐squared test (version 3.1–3; Fox and Weisberg 2019), package *performance* for model diagnostics (version 4.4.3; 2025), and package *multcomp* for multiple comparisons (version 1.4.29; Hothorn et al. 2008).

## 3 Results

The number of images recorded by camera traps under each selected tree in every plot throughout the experiment (Figure 4) exceeds the number of acorns consumed or dispersed (Figure 5), since many rodent visits did not involve acorn transport or consumption. However, the results indicate that, three rodent species (Algerian mouse, wood mouse, and garden dormouse) avoid the spots under trees with raptor models, and concentrate their activity under trees without raptor models, contrary to common voles, which, according to the number of records, make similar use of spots beneath trees with and without raptor models.

**FIGURE 4:**
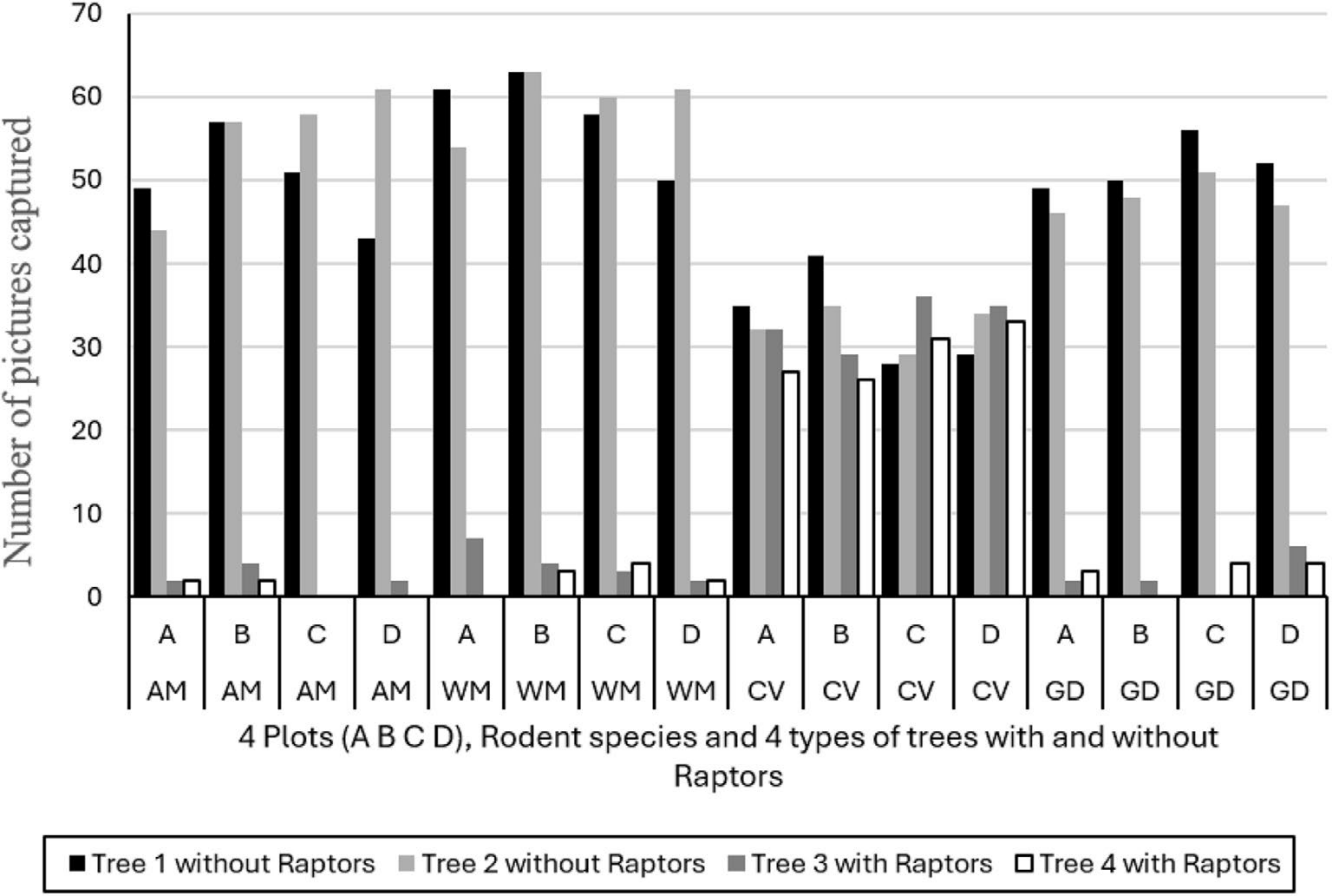
Number of pictures captured in four trees by plot (four plots). AM = Algerian mouse, CV = common vole, GD = garden dormouse, WM = Wood mouse. A = Plot 1, B = Plot 2, C = Plot 3, D = Plot 4.

**FIGURE 5:**
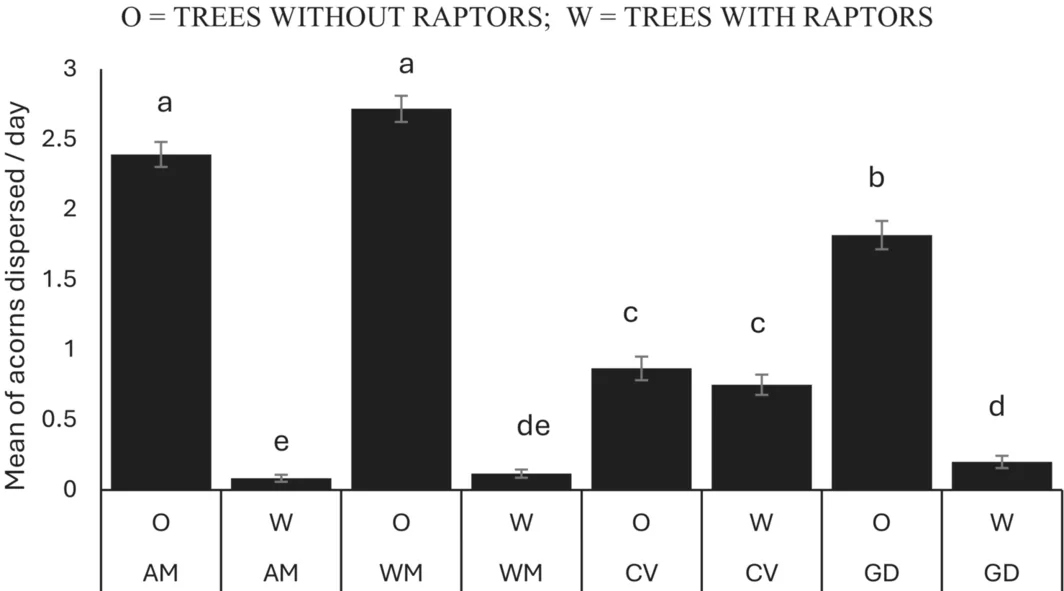
Mean number of acorns consumed or dispersed over the 15‐day by four rodent species (AM = Algerian mouse, CV = common vole, GD = garden dormouse, WM = wood mouse). Acorns were provided under trees without raptor models = O and with raptor models = W. Significant differences (*p* < 0.05) in the Benjamini–Hochberg test are indicated by different lowercase letters.

The results of the GLMM analysis in Table 1 show a significant effect of the raptor models on the number of acorns used (consumed in situ or dispersed) by rodents. The main effects of the rodent species and raptor models together with their interaction were also significant, contrary to time, the 15‐day period elapsed during the experiments, which was not significant.

**TABLE 1:** Summary results of generalized linear mixed model (GLMM) testing the effects of raptor models on the number of acorns consumed in situ or dispersed by the four rodent species over 15 days experiment. The degrees of freedom Chi‐squared values of the fixed factors and their significance (*p*) are presented.

	num df	Chisq	*p*
Number of acorns used			
Rodent	3	43.7350	< 0.0001
Raptors	1	177.0239	< 0.0001
Time	1	0.6998	0.4038
Rodent × Raptors	3	179.1167	< 0.0001
Rodent × Time	3	0.6355	0.8883
Raptors × Time	1	0.3481	0.5552
Rodent × Raptors × time	3	0.7579	0.8595

A posteriori comparison in Figure 5 shows that rodents three species (Algerian mouse, wood mouse, and garden dormouse) use many acorns from trees without raptor models but avoid trees with these models. Garden dormouse, with approximately 1.5 acorns per day, consumes or disperses significantly less acorns in absence of raptor models than Algerian mouse and wood mouse, both with more than 2 acorns per day.

Common voles used less acorns than the other three species in absence of raptor models, with less than one acorn per day, because, contrary to the other species, they do not avoid trees with raptor models.

The results of the GLMM analysis (Table 2) show significant differences in the acorns' early fates determined by the four rodent species; indeed, the analysis of the variance detected significant differences for all the effects tested with the exception of the interaction of the three factors rodent × raptors × protection/fates.

**TABLE 2:** Summary results of generalized linear mixed model (GLMM) testing the early fates of acorns (consumed in situ, moved aboveground and stored in caches or larders, differentiating unprotected and protected locations).

	num df	Chisq	*p*
Number of acorns deposited			
Rodent	20	315.215	< 0.0001
Raptors	13	275.109	< 0.0001
Protection/fates	21	600.986	< 0.0001
Rodent × Raptors	11	97.796	< 0.0001
Rodent × Protection/fates	26	388.968	< 0.0001
Raptors × Protection/fates	14	44.210	0.0550
Rodent × Raptors × Protection/fates	18	20.345	0.3137

Therefore, rodent species show clear differences in their choice of consuming acorns in situ, moving them aboveground to protected or unprotected locations, or storing them in caches or larders located in protected or unprotected areas. Significant differences also appear in acorns' early fates according to the acorn origin regarding raptor models' presence.

The early fates of acorns, whether consumed, deposited, or stored by the four rodent species, are shown in Figure 6. The figure also indicates the origin of the acorns, distinguishing those from trees with raptor models versus trees without raptors. Additionally, deposits and consumption sites are categorized according to protection: those under branches or scrub and storage or consumption sites having no aerial protection. The results indicate that Algerian mouse prefer storing acorns in larders, the artificial containers buried to mimic storage sites. Within these larders, they prefer locations under bushes having overhead protection from branches. In situ consumption is almost non‐existent in this species. A small number of acorns are deposited in caches, with a preference for sites protected by branches. A very high proportion of the acorns that this species handles and deposits come from tree canopies that do not have raptor models, since, as we have seen, its activity is reduced under canopies where raptor models are present.

**FIGURE 6:**
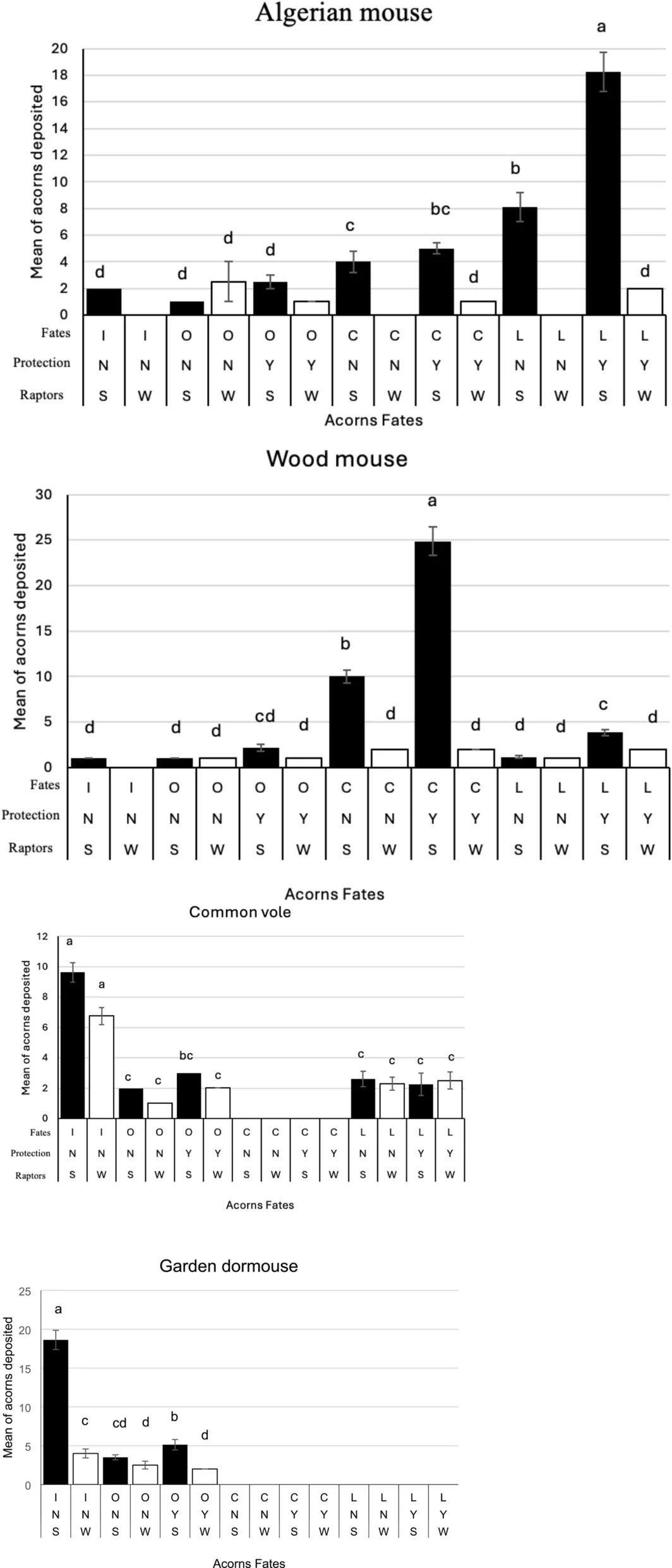
Number of acorns used or deposited by the four rodent species at different fates. Fates: I = in situ, O = on the surface, C = cache, L = larder. Protection: *N* = unprotected branch deposit, Y = deposit protected by branches. Raptors: S = acorns from tree with raptor models, W = acorns from tree without birds of raptor models. Significant differences (*p* < 0.05) of Benjamini–Hochberg comparisons are indicated by different lowercase letters.

Wood mouse, on the other hand, prefers storing acorns in caches and using those under bushes or scrub that are protected by branches. They do not consume acorns in situ and make minimal use of larders (artificial containers). In this species as well, few acorns from canopies with presence of raptor models make it to the storage sites because of the low level of activity under this type of canopy that we have already observed.

In contrast, common voles and garden dormouse consume most of their acorns in situ. There are no differences in the origin of the acorns because this species does not reduce its activity under tree canopies with raptor models. However, common voles, surprisingly, do use these containers, even though they do not typically consume this kind of food.

Garden dormouse does not store acorns in caches and does not use containers that mimic larders. However, in this species, there are differences in the source of the acorns; most of them are consumed in situ, but not those found under tree canopies where raptor models are present.

## 4 Discussion

At the outset of this study, we asked several key questions that shaped our objectives: Does the presence of birds of prey in the treetops cause rodents to avoid venturing beneath them to forage acorns? Does this reduce the number of acorns that rodents disperse?

Camera traps (Figure 4) show that three rodent species avoid tree canopies where raptor models are present (Algerian mouse, wood mouse, and garden dormouse). This forces them to alter their foraging behavior, as they do not feed on these acorns. The number of acorns consumed or dispersed (Figure 5) shows that the relationship between these rodent species and oak trees changes when raptor models are present. Rodents consume and disperse fewer acorns from tree canopies where raptor models are present. This reduction, due to the presence of these raptor models, affects the early stages of the acorn dispersal process in which some of these species (Algerian mouse, wood mouse) participate (Sivy et al. 2011; Sunyer et al. 2013; Morán‐López et al. 2022). The dispersed acorns that are not consumed in situ show that the dispersal pattern changes because of the presence of raptor models (Figure 6). Acorns from tree canopies where raptor models are present do not reach the storage sites because the dispersal species avoid venturing under these canopies to feed them. This may reduce, in the early stages, the number of acorns available for later stages (Luo et al. 2023).

Rodents play a fundamental role in the early stages of this dispersal process, as they are responsible for transporting and storing acorns, or, as acorn predators, they destroy them and halt this process. Here, we aim to determine whether the predatory relationship between rodents and birds of prey interferes with the long‐established relationship between rodents and oaks, in which certain rodent species act as collaborators by transporting and storing acorns.

The presence of these species in the tree canopies causes some rodent species to avoid these canopies by altering their foraging behavior while searching for food. By avoiding these canopies, the sources of acorns are reduced and, consequently, the number of acorns that these rodents contribute to the dispersal process in which they participate by handling the acorns in various ways (Sivy et al. 2011; Sunyer et al. 2013; Morán‐López et al. 2022). A reduction in the number of acorns reaching the storage areas, the first stage of the dispersal process, can bring the process to a standstill because not enough acorns reach the final stages of the dispersal process. However, this effect is only observed in the two species that have occupied oak habitats since the Holocene and Pleistocene (Michaux et al. 2003; Palomo et al. 2009; Domínguez García et al. 2019), Algerian mouse and wood mouse (Gray et al. 1998; Lalis et al. 2019). The behavior of avoiding trees in the presence of raptor models reduces the number of acorns that would reach the buried storage sites where the germination process, which we do not study here, would begin. In the garden dormouse, this change in behavior under the tree canopy does not affect the acorn dispersal process, as it does not participate in it, since it does not carry acorns.

In this experiment, we subjected the rodents to overexposure to the bird‐of‐prey models, as we placed four models per tree with relatively small canopies. In nature, only one bird of prey stalks a rodent at any given time due to the territorial behavior exhibited by birds of prey. Therefore, these behavioral changes in rodents may be less pronounced in nature. In any case, since we placed each model facing one of the four cardinal directions, each rodent, upon entering the canopy, perceives the presence of only one model or, at most, two. Even so, this still constitutes overexposure.

On the predation–mutualism continuum, each of the four rodent species occupies a different position based on its behavior. The common vole occupies the predation end of the continuum because it consumes acorns, destroying the embryo rather than transporting them. We have observed a scarcity of acorns that this species stores in larders, which means that it does transport some acorns, but since it opens them at the apical end (Del Arco et al. 2018; Del Arco and Del Arco 2022), it consumes the embryo first, and even if it stores acorns, they are unviable for germination. Therefore, it does not participate in the dispersal process; rather, it does not collaborate with oak species; on the contrary. The garden dormouse is also close to this end because it does not transport acorns. The Algerian mouse and the wood mouse are closer to the mutualism end because they transport, bury, and store acorns. Individuals of these species avoid entering areas lacking camouflage and hiding places (herbaceous plants and grass were removed from beneath the canopy of the four trees in each plot). One form of avoidance of risk is to act cautiously and refrain from venturing under canopies with raptors. We observed no rodent activity under these trees. Individuals of these species participate in acorn dispersal, as seen in previous studies (Del Arco et al. 2018; Del Arco and Del Arco 2022, 2023, 2025). This dispersal process, carried out intensively by these two species (Sunyer et al. 2013; Morán‐López et al. 2022), is reduced in the presence of raptors since acorns from trees with raptor models rarely reach the storage sites constructed by these species (Figure 6). These storage sites are the first steps in the dispersal process. If the number of acorns reaching these storage sites decreases because rodents do not carry them to the final stages of the process, then not enough acorns reach their destination, as we have seen in this study. Consequently, the effectiveness of acorn dispersal for oak trees may be reduced (Luo et al. 2023).

The common vole, a species that has never occupied oak habitats, doesn't avoid the treetops with models and consequently does not alter its foraging habits in their presence. Individuals engage in similar activities under tree canopies with or without the raptor models. This species does not contribute to acorn dispersal because it consumes acorns in situ destroying the embryo without transporting them. Surprisingly, acorns were found to be stored in larders by individuals of this species. This corroborates previous reports that have suggested that acorns were found in their burrows (Del Arco and Del Arco 2025). This behavior may be of concern, since by incorporating acorns in their diet, the species will ultimately destroy the seeds. This will prevent common vole from contributing to acorn dispersal. This behavior is detrimental to oak trees, as it can reduce their expansion. Common vole is more voracious than the Algerian mouse and wood mouse (Del Arco et al. 2018). This change in diet may be driven by the intense summer droughts in its current habitat and by repeated population outbreaks, which can turn the species into a pest that damages both vegetation and crops (Luque‐Larena et al. 2013). When food becomes scarce, this species is likely to seek alternative food sources. The presence of acorns in these storage sites may reflect this behavior. Furthermore, this species is a predatory consumer of acorns, devouring the embryos of the seeds (Del Arco et al. 2018; Del Arco and Del Arco 2022). Common vole individuals had no experience of predation from raptors perched on branches, since there are no trees in their natural habitat (Luque‐Larena et al. 2013). However, they may experience predation from the open sky, as birds of prey are their natural predators, so the risk perception is expected to differ from the other species. The comparison between the behavior of this species and that of the Algerian mouse and wood mouse underscores the important role of raptors in driving the behavioral changes observed in these latter species (Rosalino et al. 2013).

The garden dormouse also avoids treetops where models are present. It reduces its activity under canopies where raptor models are present. However, this species participates in the process of acorn dispersal only by moving them a short distance and leaving them on the ground, but it does not carry acorns over long distances or bury them. Its interaction with acorns is purely predatory and not collaborative; by eating the acorns whole, it destroys the embryos, as does the common vole. In these two species, the presence of raptor models does not modify their collaborative relationship with oak trees because this relationship does not exist. The garden dormouse moves fewer acorns under canopies without raptors than the Algerian mouse and the wood mouse. This may reflect its broader and more varied diet as compared to these latter species (Gil‐Delgado et al. 2010).

The fates of the acorns confirm the active participation of the Algerian mouse and the wood mouse in the acorn dispersal process, as they transport and bury acorns in different caches according to their preferences (Morán‐López et al. 2022). Algerian mouse and wood mouse prefer placing their acorn caches under the protection of branches and scrub. They probably select these locations because acorns can be processed more safely in locations that birds of prey cannot access. It is also possible that these two species do not consume acorns in situ for fear of raptors. Spending a long time under tree canopies, potentially occupied by birds of prey, is not safe for these animals. Therefore, they quickly transport acorns to safer places rather than handling them where they are found. However, the behavior of the garden dormouse does not support this hypothesis. This species avoids trees with presence of raptor models, and it reduces its activity under the canopy when raptors are present. However, it consumes the acorns in situ, spending a long time processing them until they are completely consumed. This indicates that, once the acorns have been located, this species does not appear to be concerned about potential attacks from raptor models perched on the branches. Its larger body size (250 g) and greater aggressiveness (Moreno 2002; Bertolino 2017) may provide it with a greater sense of security. However, this hypothesis will be the subject of future research, as it cannot be confirmed based on the results of this study.

## 5 Conclusions

The three rodent species (Algerian mouse, wood mouse, and garden dormouse) that inhabit oak forests avoid foraging for acorns under the canopy with raptor models. This reduces the number of acorns they process or that reach their storage sites. This is one of the first steps in the acorn dispersal process.

Garden dormice and common vole do not participate in the acorn dispersal process because they do not transport acorns and are acorn predators.

## Author Contributions

**Sergio Del Arco:** conceptualization (equal), data curation (supporting), formal analysis (equal), investigation (equal), methodology (equal), software (equal), supervision (supporting), validation (supporting), visualization (supporting). **Jose María Del Arco:** conceptualization (equal), data curation (lead), formal analysis (equal), funding acquisition (lead), investigation (equal), methodology (equal), project administration (lead), resources (lead), software (equal), supervision (equal), validation (equal), visualization (equal), writing – original draft (lead), writing – review and editing (lead).

## Funding

This study was partially supported by project «Escalera de Excelencia (CLU‐2025‐2‐07)—iuFOR Institute. “Unit of Excellence” of the University of Valladolid», funded by the Regional Government of Castilla Y León and co‐funded by the European Regional Development Fund (ERDF “Europe drives our growth”).

## Ethics Statement

All applicable international, national, and/or institutional guidelines for the care and use of animals were followed. The study was conducted according to the guidelines of the Declaration of Helsinki and approved by the Ethical Committee on Experimentation and Animal Welfare, University of Valladolid, Spain (*CEEBA*), protocol code 10307602, and date of approval February 3, 2022. All procedures involving animals in this study were carried out according to the ethical standards of the institution where the studies were conducted (*CEEBA*). The experimental procedures used were designed in accordance with the requirements of replacement, reduction, and refinement.

## Consent

All authors give their consent to participate in and publish this work.

## Conflicts of Interest

The authors declare no conflicts of interest.

## Data Availability

The datasets generated during the current study are available in the UVaDoc repository: https://uvadoc.uva.es/handle/10324/86710 and the Zenodo repository: https://doi.org/10.5281/zenodo.22872889.

## References

[ece374630-bib-0001] Antonová, K. , P. Veselý , and R. Fuchs . 2021. “Untrained Birds' Ability to Recognize Predators With Changed Body Size and Colouration in a Field Experiment.” BMC Ecology and Evolution 21: 74. 10.1186/s12862-021-01807-8. PMC8088688 33932984

[ece374630-bib-0002] Bartlett, M. S. 1937. “Properties of Sufficiency and Statistical Tests.” Proceedings of the Royal Society A: Mathematical, Physical and Engineering Sciences 160, no. 901: 268–282. 10.1098/rspa.1937.0109.

[ece374630-bib-0003] Bates, D. , M. Maechler , and B. Bolker . 2015. “lme4: Linear Mixed‐Effects Models Using S4 Classes (R Package Version 0.999999–2).” http://CRAN.R‐project.org/package=lme4.

[ece374630-bib-0004] Benjamini, Y. , and Y. Hochberg . 1995. “Controlling the False Discovery Rate: A Practical and Powerful Approach to Multiple Testing.” Journal of the Royal Statistical Society Series B: Statistical Methodology 57: 289–300. 10.1111/j.2517-6161.1995.tb02031.x.

[ece374630-bib-0005] Beránková, J. , P. Veselý , J. Sýkorová , and R. Fuchs . 2014. “The Role of Key Features in Predator Recognition by Untrained Birds.” Animal Cognition 17: 963–971. 10.1007/s10071-014-0728-1. 24458458

[ece374630-bib-0006] Bertolino, S. 2017. “Distribution and Status of the Declining Garden Dormouse *Eliomys quercinus* .” Mammal Review 47, no. 2: 133–147. 10.1111/mam.12087.

[ece374630-bib-0007] Bogdziewicz, M. , E. E. Crone , and R. Zwolak . 2020. “Do Benefits of Seed Dispersal and Caching by Scatterhoarders Outweigh the Costs of Predation? An Example With Oaks and Yellow‐Necked Mice.” Journal of Ecology 108: 10091018. 10.1111/1365-2745.13307.

[ece374630-bib-0008] Cao, L. , Z. Wang , C. Yan , J. Chen , C. Guo , and Z. Zhang . 2016. “Differential Foraging Preferences on Seed Size by Rodents Result in Higher Dispersal Success of Medium‐Sized Seeds.” Ecology 97, no. 11: 3070–3078. 10.1002/ecy.1555. 27870042

[ece374630-bib-0009] Chang, G. , and Z. Zhang . 2014. “Functional Traits Determine Formation of Mutualism and Predation Interactions in Seed‐Rodent Dispersal System of a Subtropical Forest.” Acta Oecologica 55: 43–50. 10.1016/j.actao.2013.11.004.

[ece374630-bib-0010] Cheng, J. , Y. Feng , N. Wang , et al. 2025. “Plant Diversity Affects Rodents' Selection of Different‐Sized Seeds.” Diversity 17: 643. 10.3390/d17090643.

[ece374630-bib-0011] Del Arco, J. M. , D. Beltrán , and C. Martínez‐Ruiz . 2018. “Risk for the Natural Regeneration of *Quercus* Species due to the Expansion of Rodent Species ( *Microtus arvalis* ).” Behavioral Ecology and Sociobiology 72: 1–8. 10.1007/s00265-018-2575-6.

[ece374630-bib-0012] Del Arco, J. M. , and S. Del Arco . 2022. “Partial Consumption of Acorns by Some Rodents Leads Their Relationship With Oaks Species Towards Mutualism.” Ecology and Evolutionary Biology 7, no. 1: 1–6. 10.11648/j.eeb.20220701.11.

[ece374630-bib-0013] Del Arco, S. , and J. M. Del Arco . 2023. “The Role of Partially Consumed Acorn Remains in Scatter Hoards and Their Implications in Oak Colonization.” Behavioral Ecology and Sociobiology 77: 132. 10.1007/s00265-023-03409-4.

[ece374630-bib-0014] Del Arco, S. , and J. M. Del Arco . 2025. “How Rodent Species Adapt to the Food Resources of Their Habitat.” Animals 15, no. 13: 1874. 10.3390/ani15131874. PMC12248874 40646773

[ece374630-bib-0015] Domínguez García, A. C. , C. Laplana , P. Sevilla , H.‐A. Blain , N. Palomares Zumajo , and L. Benítez de Lugo Enrich . 2019. “New Data on the Introduction and Dispersal Process of Small Mammals in Southwestern Europe During the Holocene: Castillejo del Bonete Site (Southeastern Spain).” Quaternary Science Reviews 225: 106008.

[ece374630-bib-0016] Fedyń, I. , T. Figarski , and L. Kajtoch . 2021. “Overview of the Impact of Forest Habitats Quality and Landscape Disturbances on the Ecology and Conservation of Dormice Species.” European Journal of Forest Research 140: 511–526. 10.1007/s10342-021-01362-3.

[ece374630-bib-0017] Fox, J. , and S. Weisberg . 2019. An R Companion to Applied Regression. 3rd ed. Sage.

[ece374630-bib-0018] Galetti, M. , R. Guevara , L. A. Galbiati , C. L. Neves , R. R. Rodarte , and C. P. Mendes . 2015. “Seed Predation by Rodents and Implications for Plant Recruitment in Defaunated Atlantic Forests.” Biotropica 47: 521–525. 10.1111/btp.12246.

[ece374630-bib-0019] Gelman, A. , and J. Hill . 2007. Data Analysis Using Regression and Multilevel/Hierarchical Models. Cambridge University Press.

[ece374630-bib-0020] Gil‐Delgado, J. A. , O. Mira , A. Viñals , J. Gómez , N. Banyuls , and C. Vives‐Ferrando . 2010. “Diet of the Garden Dormouse ( *Eliomys quercinus* Linnaeus 1766) in Orange Groves: Seasonal Variation and Use of Available Resources.” Mammalia 74: 147–151. 10.1515/MAMM.2010.027.

[ece374630-bib-0021] Gleditsch, J. M. , A. M. Hruska , and J. T. Foster . 2017. “Connecting Resource Tracking by Frugivores to Temporal Variation in Seed Dispersal Networks.” Ecology and Evolution 5: 98. 10.3389/fevo.2017.00098.

[ece374630-bib-0022] Gómez, J. M. , E. W. Schupp , and P. Jordano . 2019. “Synzoochory: The Ecological and Evolutionary Relevance of a Dual Interaction.” Biological Reviews 94: 874–902. 10.1111/brv.12481. 30467946

[ece374630-bib-0023] Gray, S. J. , J. L. Hurst , R. Stidworthy , J. Smith , R. Preston , and R. MacDougall . 1998. “Microhabitat and Spatial Dispersion of the Grassland Mouse ( *Mus spretus* Lataste).” Journal of Zoology 246: 299–308. 10.1017/s0952836998009947.

[ece374630-bib-0024] Gu, H. , Q. Zhao , and Z. Zhang . 2017. “Does Scatter‐Hoarding of Seeds Benefit Cache Owners or Pilferers?” Integrative Zoology 12: 477–488. 10.1111/1749-4877.12274. PMC5725728 28688134

[ece374630-bib-0065] Hothorn, T. , F. Bretz , and P. Westfall . 2008. “Simultaneous Inference in General Parametric Models.” Biometrical Journal 50, no. 3: 346–363. 10.1002/bimj.200810425 18481363

[ece374630-bib-0025] Hou, L. , H. Dou , M. Hu , and L. Cao . 2024. “Effects of Cache Distribution and Size on Cache Foraging by Animals.” Animal Behaviour 211: 91–98. 10.1016/j.anbehav.2024.03.005.

[ece374630-bib-0026] Lalis, A. , T. Mona , E. Stoetzel , et al. 2019. “Out of Africa: Demographic and Colonization History of the Algerian Mouse ( *Mus spretus* Lataste).” Heredity 122: 150–171. 10.1038/s41437-018-0089-7 PMC6327062 29795180

[ece374630-bib-0027] Lichti, N. I. , M. A. Steele , and R. K. Swihar . 2017. “Seed Fate and Decision‐Making Processes in Scatter‐Hoarding Rodents.” Biological Reviews 92: 474–504. 10.1111/brv.12240. 26587693

[ece374630-bib-0028] Luo, Y. , J. Cheng , X. Yan , et al. 2023. “Density‐Dependent Seed Predation of *Quercus wutaishanica* by Rodents in Response to Different Seed States.” Animals 13: 1732. 10.3390/ani13111732. PMC10251812 37889661

[ece374630-bib-0029] Luque‐Larena, J. J. , F. Mougeot , J. Viñuela , et al. 2013. “Recent Large‐Scale Range Expansion and Outbreaks of the Common Vole ( *Microtus arvalis* ) in NW Spain.” Basic and Applied Ecology 14: 432–441. 10.1016/j.baae.2013.04.006.

[ece374630-bib-0030] Michaux, J. R. , E. Magnanou , E. Paradis , C. Nieberding , and R. Libois . 2003. “Mitochondrial Phylogeography of the Wood Mouse ( *Apodemus sylvaticus* ) in the Western Paleoartic Region.” Molecular Ecology 12: 685–697. 10.1046/j.1365-294x.2003.01752.x 12675824

[ece374630-bib-0031] Mittelman, P. , A. S. Pires , and F. A. S. Fernandez . 2021. “The Intermediate Dispersal Hypothesis: Seed Dispersal Is Maximized in Areas With Intermediate Usage by Hoarders.” Plant Ecology 222: 221–231. 10.1007/s11258-020-01100-6.

[ece374630-bib-0032] Moore, C. M. , and J. W. Dittel . 2020. “Seed‐Dispersing Animals on the Plants They Disperse.” Journal of Ecology 108: 1775–1783. 10.1111/1365-2745.13414.

[ece374630-bib-0033] Morán‐López, T. , J. Sánchez‐Dávila , I. Torre , A. Navarro‐Castilla , I. Barja , and M. Díaz . 2022. “Ungulate Presence and Predation Risks Reduce Acorn Predation by Mice in Dehesas.” PLoS One 17, no. 8: e0260419. 10.1371/journal.pone.0260419. PMC9377575 35969588

[ece374630-bib-0034] Moreno, S. 2002. “Lirón Careto *Eliomys quercinus* (LINNAEUS, 1766).” Galemys 14, no. 1: 1–16.

[ece374630-bib-0035] Mulungu, E. L. , and M. J. Martin . 2024. “Fight or Flight? Responses of Rodents to Predator Cues: A Review.” Journal of Current Opinion in Crop Science 5, no. 3: 191–204. 10.62773/jcocs.v5i3.260.

[ece374630-bib-0036] Muñoz, A. , and R. Bonal . 2011. “Linking Seed Dispersal to Cache Protection Strategies.” Journal of Ecology 99: 1016–1025. 10.1111/j.1365-2745.2011.01818.x.

[ece374630-bib-0037] Palomo, L. J. , E. R. Justo , and J. M. Vargas . 2009. “ *Mus spretus* (Rodentia: Muridae).” Mammalian Species 840: 1–10. 10.1644/840.1.

[ece374630-bib-0039] R‐Core Team . 2025. R: A Language and Environment for Statistical Computing. R Foundation for Statistical Computing. http://www.R‐project.org/.

[ece374630-bib-0040] Rosalino, L. M. , F. Nóbrega , M. Santos‐Reis , G. Teixeira , and R. Rebelo . 2013. “Acorn Selection by the Wood Mouse *Apodemus sylvaticus* : A Semi‐Controlled Experiment in a Mediterranean Environment.” Zoological Science 30, no. 9: 724–730. 10.2108/zsj.30.724. 24004078

[ece374630-bib-0041] Sivy, K. J. , S. M. Ostoja , E. W. Schupp , and S. Durham . 2011. “Effects of Rodent Species, Seed Species, and Predator Cues on Seed Fate.” Acta Oecologica 37: 321–328. 10.1016/j.actao.2011.03.004.

[ece374630-bib-0042] Snell, R. S. , N. G. Beckman , E. Fricke , et al. 2019. “Consequences of Intraspecific Variation in Seed Dispersal for Plant Demography, Communities, Evolution and Global Change.” AoB Plants 11: plz016. 10.1093/aobpla/plz016. PMC6644487 31346404

[ece374630-bib-0043] Steele, M. A. 2021. Oak Seed Dispersal: A Study in Plant‐Animal Interaction. Johns Hopkins University Press.

[ece374630-bib-0044] Steele, M. A. , M. Bugdal , A. Yuan , et al. 2011. “Cache Placement, Pilfering, and a Recovery Advantage in a Seed‐Dispersing Rodent: Could Predation of Scatter Hoarders Contribute to Seedling Establishment? (Special Issue: Frugivores and Seed Dispersal: Mechanisms and Consequences of a Key Interaction for Biodiversity).” Acta Oecologica 37, no. 6: 554–560.

[ece374630-bib-0045] Steele, M. A. , T. Knowles , K. Bridle , and E. L. Simms . 1993. “Tannins and Partial Consumption of Acorns: Implications for Dispersal of Oaks by Seed Predators.” American Midland Naturalist 130: 229–238. 10.2307/2426123.

[ece374630-bib-0046] Sunyer, P. , A. Muñoz , R. Bonal , and J. M. Espelta . 2013. “The Ecology of Seed Dispersal by Small Rodents: A Role for Predator and Conspecific Scents.” Functional Ecology 27: 1313–1321. 10.1111/1365-2435.12143.

[ece374630-bib-0047] Vander Wall, S. B. 1990. Food Hoarding in Animals. University of Chicago Prees.

[ece374630-bib-0048] Vander Wall, S. B. 2010. “How Plants Manipulate the Scatter‐Hoarding Behaviour of Seed‐Dispersing Animals.” Philosophical Transactions of the Royal Society, B: Biological Sciences 365: 989–997. 10.1098/rstb.2009.0205. PMC2830241 20156821

[ece374630-bib-0049] Wang, B. , J. Chen , and R. T. Corlett . 2014. “Factors Influencing Repeated Seed Movements by Scatter‐Hoarding Rodents in an Alpine Forest.” Scientific Reports 4: 1–6. 10.1038/srep04786. PMC3998018 24759374

[ece374630-bib-0050] Wang, B. , and R. T. Corlett . 2017. “Scatter‐Hoarding Rodents Select Different Caching Habitats for Seeds With Different Traits.” Ecosphere 8, no. 4: e01774. 10.1002/ecs2.1774.

[ece374630-bib-0051] Wang, Z. , L. Cao , and Z. Zhang . 2014. “Seed Traits and Taxonomic Relationships Determine the Occurrence of Mutualisms *Versus* Seed Predation in a Tropical Forest Rodent and Seed Dispersal System.” Integrative Zoology 9: 309–319. 10.1111/1749-4877.12086. 24382317

[ece374630-bib-0052] Wang, Z. , B. Wang , C. Yan , S. Yuan , and L. Cao . 2020. “Neighborhood Effects on the Tannin‐Related Foraging Decisions of Two Rodent Species Under Semi‐Natural Conditions.” Integrative Zoology 15: 569–577. 10.1111/1749-4877.12473. 32645258

[ece374630-bib-0053] Wróbel, A. , and R. Zwolak . 2017. “Deciphering the Effects of Disperser Assemblages and Seed Mass on Patterns of Seed Dispersal in a Rodent Community.” Integrative Zoology 12: 457–467. 10.1111/1749-4877.12265. 28488781

[ece374630-bib-0054] Xiao, Z. , and C. J. Krebs . 2015. “Modeling the Costs and Benefits of Seed Scatterhoarding to Plants.” Ecosphere 6: 1–8. 10.1890/ES14-00438.1.

[ece374630-bib-0055] Yi, X. , and Z. Wang . 2015. “Context‐Dependent Seed Dispersal Determines Acorn Survival of Sympatric Oak Species.” Plant Ecology 216: 123–132. 10.1007/s11258-014-0421-0.

[ece374630-bib-0056] Zhang, H. , Z. Wang , Q. Zeng , and G. Chang . 2015. “Mutualistic and Predatory Interactions Are Driven by Rodent Body Size and Seed Traits in a Rodent–Seed System in Warm‐Temperate Forest in Northern China.” Wildlife Research 42: 149–157. 10.1071/wr14211.

[ece374630-bib-0057] Zhang, H. , C. Yan , G. Chang , and Z. Zhang . 2016. “Seed Trait‐Mediated Selection by Rodents Affects Mutualistic Interactions and Seedling Recruitment of Co‐Occurring Tree Species.” Oecologia 180: 475–484. 10.1007/s00442-015-3490-4. 26546082

[ece374630-bib-0058] Zhang, H. , and Z. Zhang . 2008. “Endocarp Thickness Affects Seed Removal Speed by Small Rodents in a Warm‐Temperate Broad‐Leafed Deciduous Forest, China.” Acta Oecologica 34: 285–293. 10.1016/j.actao.2008.06.001.

[ece374630-bib-0059] Zhang, J. , X. Yan , B. Dayananda , J. Cheng , and Y. Luo . 2024. “Frequency‐Dependent Seed Selection by Rodents: Response to Seed Tannins and Sizes.” Global Ecology and Conservation 54: e03073.

[ece374630-bib-0060] Zhang, M. , M. A. Steele , and X. Yi . 2013. “Reconsidering the Effects of Tannin on Seed Dispersal by Rodents: Evidence From Enclosure and Field Experiments With Artificial Seeds.” Behavioural Processes 100: 200–207. 10.1016/j.beproc.2013.09.010. 24161819

[ece374630-bib-0061] Zhang, Y. , A. W. Bartlow , Z. Wang , and X. Yi . 2018. “Effects of Tannins on Population Dynamics of Sympatric Seed‐Eating Rodents: The Potential Role of Gut Tannin‐Degrading Bacteria.” Oecologia 187: 667–678. 10.1007/s00442-018-4151-1. 29736862

[ece374630-bib-0062] Zhang, Y. , M. Holyoak , Z. Zhang , J. Chen , and C. Chuan Yan . 2025. “The Network Architecture and Phylogeographic Drivers of Interactions Between Rodents and Seed Plants at Continental Scales.” Journal of Animal Ecology 94: 760–773. 10.1111/1365-2656.70013 39967252

[ece374630-bib-0063] Zwolak, R. , M. Bogdziewicz , and E. E. Crone . 2020. “On the Need to Evaluate Costs and Benefits of Synzoochory for Plant Populations.” Journal of Ecology 108: 1784–1788. 10.1111/1365-2745.13463.

[ece374630-bib-0064] Zwolak, R. , and E. E. Crone . 2012. “Quantifying the Outcome of Plant–Granivore Interactions.” Oikos 121: 20–27. 10.1111/j.1600-0706.2011.019849.x.

